# Parental Death during Childhood and Adult Cardiovascular Risk in a Developing Country: The Guangzhou Biobank Cohort Study

**DOI:** 10.1371/journal.pone.0019675

**Published:** 2011-05-16

**Authors:** C. Mary Schooling, ChaoQiang Jiang, Tai Hing Lam, WeiSen Zhang, Kar Keung Cheng, Gabriel M. Leung

**Affiliations:** 1 Department of Community Medicine, School of Public Health, The University of Hong Kong, Hong Kong SAR, China; 2 Guangzhou Occupational Diseases Prevention and Treatment Centre, Guangzhou Number 12 Hospital, Guangzhou, China; 3 Department of Public Health and Epidemiology, University of Birmingham, Birmingham, United Kingdom; 4 School of Public Health, The City University of New York (CUNY), New York, New York, United States of America; University of Michigan, United States of America

## Abstract

**Background:**

In observational studies from western countries childhood emotional adversity is usually associated with adult cardiovascular disease. These findings are open to contextual biases making evidence from other settings valuable. We examined the association of a potential marker of childhood emotional adversity with cardiovascular disease risk factors in a developing country.

**Methods:**

We used multivariable regression in cross-sectional analysis of older (≥50 years) men (n = 7,885) and women (n = 20,886) from the Guangzhou Biobank Cohort Study (2003–8) to examine the adjusted association of early life (<18 years) parental death (none, one or two deaths) with blood pressure, fasting glucose, LDL-cholesterol, HDL-cholesterol, triglycerides, body mass index (BMI), waist-hip ratio (WHR) and white blood cell count (WBC). We used seated height and delayed 10-word recall to assess content validity of parental death as a measure of childhood emotional adversity. We also examined whether associations varied by sex.

**Results:**

Early life parental death was associated with shorter age- and sex-adjusted seated height. It was also associated with lower 10-word recall score adjusted for age, sex, socio-economic position, leg length and lifestyle. Similarly, adjusted early life parental death was not associated with blood pressure, fasting glucose, LDL-cholesterol or HDL-cholesterol but was associated with lower BMI (−0.40, 95% confidence interval (CI) −0.62 to −0.19 for 2 compared with no early life parental deaths) and triglycerides. Associations varied by sex for WHR and WBC. Among men only, early life parental death was associated with lower WHR (−0.008, 95% CI −0.015 to −0.001) and WBC (−0.35 10^9^/L, 95% CI −0.56 to −0.13).

**Conclusions:**

In a non-western population from a developing country, childhood emotional adversity was negatively associated with some cardiovascular risk factors, particularly among men. Our study suggests that some of the observed associations in western populations may be socially rather than biologically based or may be population specific.

## Introduction

Low lifetime and childhood socio-economic position (SEP) is associated with cardiovascular disease [1] and its risk factors [2], [3]. Low childhood socio-economic position may encompass economic adversity, such as poor nutrition and lack of access to facilities, as well as emotional adversity, encompassing emotionally distressing childhood or adolescent experiences, such as parental absence, neglect or maltreatment. Early life emotional adversity may make a contribution to adult cardiovascular disease in addition to the long-term biological effects of early life economic adversity. Early life emotional adversity may also be a pathway underlying the association of low childhood SEP with cardiovascular disease [4]. Observations from western populations suggest that early life emotional adversity is associated with adult cardiovascular diseases [5]–[10]. However in these populations there are also associations between childhood economic adversity and subsequent biological cardiovascular risk [1]. Adverse economic and emotional conditions tend to co-occur [11], [12] making examination of the role of emotional adversity in such populations open to residual confounding.

To the best of our knowledge the association between early life emotional adversity and cardiovascular risk has not been examined in a developing country, although such populations provide a different context in which to check the reproducibility of associations observed in western populations in the absence of experimental evidence. A long-running cohort study from a developing country with contemporaneous recording of childhood emotional state would enable prospective examination of this question and would avoid the use of retrospective reporting of childhood emotional adversity which may be affected by current emotional state. Historical social and economic circumstances in developing countries preclude the existence of such long-running cohort studies. However, for adults in developing countries parental death during childhood is not uncommon. Parental death is one of the most significant stressors a child can experience [13]–[15]. Age at parental death is a notable event and unlikely to be influenced by current emotional state. We took advantage of a large study from economically developing southern China to assess the association of early life parental death with adult cardiovascular risk factors. We considered traditional cardiovascular risk as reflected in the Framingham score or the metabolic syndrome, i.e., blood pressure, LDL-cholesterol, HDL-cholesterol, fasting glucose, triglycerides and adiposity, proxied by body mass index and waist-hip ratio [16]. We also considered non-traditional risk factors, such as markers of inflammation, as these have also been observed to be associated childhood emotional adversity [4] and with cardiovascular risk [17], [18]. Here, we used white cell count as a marker of inflammation because it is the only commonly used marker of inflammation available for most of the participants. Finally, we also used seated height and cognitive status as outcomes to check the validity of using early life parental death as a measure of early life emotional adversity, because sustained stress in childhood would be expected to reduce seated height [19] and adversely affect cognition [20].

## Methods

### Ethics Statement

The Guangzhou Medical Ethics Committee of the Chinese Medical Association approved the study and all participants gave written, informed consent before participation.

### Sources of data

The Guangzhou Biobank Cohort Study is a collaboration between the Guangzhou No. 12 Hospital, the Universities of Hong Kong and Birmingham, and has been described in detail elsewhere [21]. Recruitment of participants draws from “The Guangzhou Health and Happiness Association for the Respectable Elders”, a community social and welfare association unofficially aligned with the municipal government where membership is open to anyone aged 50 years or older for a monthly, nominal fee of 4 Yuan (50 US cents). Participants were recruited into the study in three recruitment phases. Recruitment for phase 1 took place in September 2003 to September 2004, for phase 2 from April 2005 to May 2006 and for phase 3 from September 2006 to January 2008. About 7% of permanent Guangzhou residents aged 50 years and over are members of “The Guangzhou Health and Happiness Association for the Respectable Elders”, of whom 33% enrolled for recruitment phases 1, 2 or 3, and were included if they were capable of consenting, ambulatory, and not receiving treatment modalities which if omitted may result in immediate life threatening risk, such as chemotherapy or radiotherapy for cancer, or dialysis for renal failure. Participants underwent a half-day detailed medical interview, including disease history, and physical examination.

The detailed methods of measurement have been reported elsewhere [21]. In brief, standing height was measured without shoes to the nearest 0.1 centimeter. Sitting height was measured with the participants sitting on a standard stool; leg length was calculated as the difference between height and sitting height. Weight was measured in light clothing to the nearest 0.1 kilogram. Hip circumference was measured at the greatest circumference round the buttocks below the iliac crest. Waist circumference was measured horizontally around the smallest circumference between the ribs and iliac crest, or at the level of the navel for obese participants. Seated blood pressure was recorded as the average of the last two of three measurements, using the Omron 705CP sphygmomanometer. Lipids and glucose were determined by the Shimadzu CL-8000 Clinical Chemical Analyzer. White blood cell count was determined using a SYSMEX KX-21 haematology analyser.

### Exposure assessment

Early life parental death was classified on a three point scale as no parental death before adulthood, death of one parent before adulthood and death of both parents before adulthood. As is common in most settings we defined adult as ≥18 years, because by this age young people are relatively self-sufficient, and so might be less affected by parental death. Moreover the period of plasticity for the stress system and particular sensitivity to stress with potential long-term consequences may extend from infancy through adolescence [22], [23]. We also considered a supplementary categorization according to age, i.e., earliest death of a parent at ages 0–<3 years, 3–<13 years, 13–<18 years and 18+ years.

To assess content and predictive validity, we used seated height and cognitive status, proxied by delayed 10-word recall score, as validation outcomes, which even after adjustment for confounding would be expected to be negatively associated with sustained or chronic early life stress. Early life stress has previously been observed to impair seated height [19]. Sustained or chronic early life stress would also be expected to impair cognition [20], [24] by detrimentally affecting the development of the hippocampus [25]. The adapted 10-word list learning task has been validated as a culturally and educationally sensitive tool for identifying dementia in population based research in developing countries [26]. The validation outcomes were considered as continuous quantities in the units in which they were measured, they were not standardized.

### Outcome measures

Nine biological outcomes were used. Eight correspond to well-established predictors of cardiovascular risk as in the Framingham score or the metabolic syndrome [16], i.e., systolic blood pressure, diastolic blood pressure, fasting plasma glucose, HDL-cholesterol, LDL-cholesterol, triglycerides, waist-hip ratio and body mass index. We also included white blood cell count because it is associated with cardiovascular risk [17], [18], and the detrimental impact of early life emotional adversity may operate via permanent alteration of the stress response [4], reducing glucocorticoid signaling and resulting in a pro-inflammatory state [4]. White cell count was only available for recruitment phases 1 and 3. These outcomes were considered as continuous quantities in the units in which they were measured, they were not standardized.

### Statistical analysis

Multivariable censored linear regression was used to assess the association of parental death with blood pressure, lipids and fasting glucose because some people were taking medications for hypertension (n = 6,473), hyperlipidemia (n = 1,820) or diabetes (n = 2,181). These models censored the outcome for those on medication at the observed value so that the true measurement for blood pressure, LDL-cholesterol, triglycerides and fasting plasma glucose were assumed to be that observed or higher, whilst the true measurement for HDL-cholesterol was assumed to be that observed or lower. Multivariable linear regression was used for body mass index, waist-hip ratio, white blood cell count and the validation outcomes. From these models we present beta coefficients with 95% confidence intervals. The beta coefficient represents the adjusted mean difference in the outcome, in the units in which it was measured, relative to the reference category. We examined whether the outcomes had different associations with parental death by sex or age from the heterogeneity across subgroups and the p-values of the relevant interaction terms.

Potential confounders considered were age (in 5 year age-groups), socio-economic position (father's job, own education and own longest held occupation), a measure of childhood living conditions (leg length) and lifestyle habits (smoking, use of alcohol and physical activity) categorized as in Table 1. Where any of father's job (38%), longest held occupation (13%) or early life parental death (7%) were not available we used multiple imputation (10 imputations), which uses all available data, preserves uncertainty from missing data [27], minimizes inclusion bias and increases statistical power [28]. Missing values were predicted based on a flexible additive regression model with predictive mean matching [29] incorporating data on age, sex, leg length, education, parental death, father's job, longest held occupation and all the outcomes [30] (systolic blood pressure, diastolic blood pressure, fasting plasma glucose, HDL-cholesterol, LDL-cholesterol, triglycerides, waist-hip ratio and body mass index, seated height and 10-word recall score) except white blood cell count because white blood cell count is only available for two of the three recruitment phases. We imputed missing values once only and analyzed the one set of ten complete imputed datasets separately for each outcome. We summarized the results into single estimated beta-coefficients for each outcome with confidence intervals and p-values adjusted for the missing data uncertainty [28].

**Table 1:** Characteristics by early life parental death in 26,820 older Chinese men and women in recruitment phases 1, 2 and 3 of The Guangzhou Biobank Cohort Study (2003–8).

		Men			Women		
		Parental deaths before adulthood (before age of 18 years)			Parental deaths before adulthood (before age of 18 years)		
		None	One	Two	None	One	Two
N		5025	2088	360	14099	4,624	624
Age (years)	Mean and SD	63.0 (6.7)	65.8 (6.3)	68.2 (5.6)	59.5 (6.6)	63.2 (7.1)	66.4 (6.3)
Father's job‡	Manual	52.2	53.2	51.9	48.6	51.2	48.4
	Non-manual	13.3	9.1	8.9	13.5	11.3	9.6
	Unknown	34.5	37.7	39.2	37.9	37.6	42.0
Education	Less than primary	1.4	3.4	8.9	8.6	18.0	34.6
	Primary	22.9	33.4	35.0	32.4	40.0	39.6
	Junior middle	30.8	28.3	26.4	27.8	22.5	13.0
	Senior middle	26.4	19.5	14.7	24.5	15.0	9.5
	Junior college	10.3	8.6	7.2	4.8	2.9	2.6
	College	8.2	6.9	7.8	1.9	1.5	0.6
Longest held	Manual	50.0	52.5	56.4	65.0	70.4	74.7
occupation ‡	Non-manual	38.1	38.0	36.4	19.9	16.4	14.3
	Unknown	11.9	9.5	7.2	15.1	13.2	11.1
Smoking status	Never	40.9	38.4	39.7	97.0	95.5	93.4
	Ex-smoker	27.1	31.3	32.2	1.4	2.0	3.2
	Current	31.9	30.2	28.1	1.6	2.5	3.2
Alcohol use	Never	53.0	53.4	51.9	78.2	80.6	79.0
	<1/week	23.0	21.7	21.4	16.7	13.9	13.6
	1–4/week	6.5	5.2	6.1	1.6	1.6	2.1
	5+/week	10.9	13.0	13.9	1.4	1.6	2.2
	Ex-drinker	5.0	5.6	4.7	2.2	2.3	1.9
	unknown	1.6	1.1	1.9	1.7	1.3	1.1
Physical activity	Inactive	9.0	8.0	3.1	8.4	7.2	6.4
(IPAQ)	Minimally active	45.6	42.2	45.0	39.1	40.2	41.5
	HEPA† active	45.5	49.8	51.9	52.5	52.6	52.1

†health enhancing physical activity, i.e. vigorous activity at least 3 days a week achieving at least 1500 MET minutes per week or activity on 7 days of the week achieving at least 3000 MET minutes per week.

‡Manual occupations are agricultural worker, factory work or sales and service; non-manual are administrator/manager, professional/technical, military/disciplined.

Model 1 adjusted for age and also for sex and the interaction of age and sex when men and women were considered together. Model 2 additionally adjusted for life course socio-economic position, a proxy of early living conditions (leg length) [19], [31] and lifestyle. Model 3 additionally adjusted the cardiovascular risk factor outcomes for body mass index and waist-hip ratio. Analysis was carried out using R version 2.12.1 (R Development Core Team, Vienna, Austria).

## Results

Of the 30,499 participants recruited in phases 1, 2 or 3, 28,771 (94.3%) had complete data on all outcomes, and 26,820 had age at parental death. There were more women (20,886) than men (7,885), and the women were younger (mean age 60.9 (standard deviation (SD) 7.08)) than the men (mean age 64.2 (SD 6.74)). Age ranged from 50 to 96 years, but only 668 participants were older than 75 years.

Table 1 shows the associations of early life parental death with potential confounders without imputation. Early life parental death was associated with older age, less education, manual occupation (of self and father), regular alcohol use and more physical activity. As is typical in this setting, relatively few participants used alcohol most days of the week or were current smokers, particularly amongst the women.

Table 2 shows the association of early life parental death with the validation outcomes and pre-specified cardiovascular risk factors after imputation, 25.4% (7,300) had one parental death before 18 years and 3.4% (1,094) had two parental deaths before 18 years. Early life parental death was associated with lower seated height and 10-word recall score, as expected, with no evidence of differences by sex. For men and women together, early life parental death had little association with blood pressure, fasting glucose, HDL-cholesterol, LDL-cholesterol or waist-hip ratio, but was associated with lower body mass index and with lower triglycerides (p-value for trend 0.01), with no evidence of different associations by age (data not shown). Estimates for the cardiovascular outcomes were generally similar in all three models, i.e., adjusted for age, sex and the interaction of age and sex (model 1), additionally adjusted for socio-economic position, leg length and lifestyle (model 2) or further adjusted for body mass index and waist-hip ratio (model 3) if appropriate. Adjustment for confounders (model 2 compared with model 1) strengthened the negative association of early life parental death with body mass index. However, adjustment for body mass index and waist-hip ratio (model 3 compared with model 2) attenuated the association of early life parental death with triglycerides.

**Table 2:** Adjusted associations of early life parental death with cardiovascular risk factors, seated height and delayed 10-word recall score in 28,771 older Chinese men and women in recruitment phases 1, 2 and 3 of The Guangzhou Biobank Cohort Study (2003–8).

	Mean and standard deviation			Parental deaths before adulthood(before age of 18 years)					p-value for interaction by sex
			Model †	None	One parent		Two parents		
	Men	Women			β	95% CI	β	95% CI	
Systolic blood	133.3	129.2	1	ref	0.30	−0.42 to 1.08	0.35	−1.47 to 2.18	0.07
pressure (mm Hg)	±21.5	±22.2	2	ref	−0.06	−0.78 to 0.67	−0.29	−2.10 to 1.53	0.09
			3	ref	0.24	−0.45 to 0.94	0.61	−1.13 to 2.35	0.16
Diastolic blood	76.3	72.6	1	ref	−0.05	−0.42 to 0.33	−0.23	−1.14 to 0.69	0.07
pressure (mm Hg)	±11.3	±11.0	2	ref	−0.16	−0.54 to 0.21	−0.43	−1.33 to 0.48	0.08
			3	ref	0.01	−0.35 to 0.37	0.08	−0.79 to 0.95	0.14
Fasting plasma	5.72	5.75	1	Ref	0.00	−0.05 to 0.05	0.02	−0.09 to 0.14	0.11
glucose (mmol/L)	1.54	±1.70	2	Ref	−0.01	−0.06 to 0.04	0.003	−0.11 to 0.12	0.12
			3	ref	−0.002	−0.05 to 0.05	0.04	−0.07 to 0.15	0.23
LDL-cholesterol	3.07	3.34	1	ref	−0.02	−0.04 to 0.004	−0.01	−0.06 to 0.04	0.82
(mmol/L)	±0.65	±0.71	2	ref	−0.01	−0.04 to 0.01	−0.00	−0.05 to 0.05	0.71
			3	ref	−0.01	−0.03 to 0.01	0.01	−0.04 to 0.05	0.79
HDL-cholesterol	1.52	1.71	1	ref	0.00	−0.01 to 0.01	0.02	−0.01 to 0.04	**0.04**
(mmol/L)	±0.38	±0.40	2	ref	0.00	−0.01 to 0.01	0.02	−0.01 to 0.04	0.06
			3	ref	0.00	−0.02 to 0.01	0.00	−0.02 to 0.03	0.15
Triglycerides	1.65	1.69	1	ref	**−0.04**	**−0.08 to −0.001**	−0.08	−0.17 to 0.005	0.90
(mmol/L)	±1.30	±1.24	2	ref	**−0.04**	**−0.08 to −0.004**	−0.09	−0.18 to 0.001	0.86
			3	ref	−0.03	−0.07 to 0.004	−0.05	−0.13 to 0.04	0.49
Body mass index	23.5	23.9	1	ref	**−0.11**	**−0.21 to −0.02**	**−0.27**	**−0.48 to −0.05**	0.43
	±3.2	±3.4	2	ref	**−0.17**	**−0.27 to −0.08**	**−0.40**	**−0.62 to −0.19**	0.52
Waist-hip ratio	0.90	0.85	1	ref	0.001	−0.001 to 0.003	−0.003	−0.007 to 0.001	**0.02**
	±0.06	±0.07	2	ref	−0.001	−0.002 to 0.001	**−0.006**	**−0.01 to −0.002.004**	**0.03**
White blood cell	6.77	6.36	1	ref	0.01	−0.04 to 0.07	0.01	−0.12 to 0.13	**0.01**
count * (10^9^/L)	±1.68	±1.55	2	ref	−0.03	−0.09 to 0.03	−0.08	−0.20 to 0.04	**0.01**
			3	ref	−0.02	−0.08 to 0.03	−0.03	−0.15 to 0.09	**0.01**
Delayed 10-word	5.3	5.57	1	ref	**−0.17**	**−0.22 to −0.12**	**−0.40**	**−0.51 to −0.28**	0.20
recall score	±1.79	±1.87	2	ref	**−0.08**	**−0.13 to −0.03**	**−0.21**	**−0.32 to −0.10**	0.40
Seated height	88.6	83.4	1	ref	**−0.32**	**−0.41 to −0.22**	**−0.86**	**−1.08 to −0.65**	0.50
(centimeters)	±3.4	±3.4	2	ref	**−0.18**	**−0.27 to −0.08**	**−0.59**	**−0.81 to −0.38**	0.55

†Model 1 adjusted for age, sex and the interaction of age and sex.

Model 2 additionally adjusted for father's job type, leg length, education, job type, smoking, use of alcohol and physical activity.

Model 3 additionally adjusted for body mass index and waist-hip ratio.

*only available in recruitment phases 1 and 3 for 5285 men and 14150 women.

**Bold type indicates statistical significance.**

There was some evidence of different associations by sex for HDL-cholesterol, waist-hip ratio and white blood cell count, for which Table 3 shows sex-specific associations. Table 3 shows that early life parental death was associated with lower waist-hip ratio and white cell count among men. Estimates were generally similar in all models, i.e., adjusted for age (model 1), additionally adjusted for socio-economic position, leg length and lifestyle (model 2) or further adjusted for body mass index and waist-hip ratio (model 3) if appropriate. However, adjustment for confounders (model 2 compared with model 1) attenuated the association of early life parental death with higher white cell count among women.

**Table 3:** Sex-specific adjusted associations of early life parental death with HDL-cholesterol, waist-hip ratio and white blood cell count in 28,771 older Chinese men and women in recruitment phases 1, 2 and 3 of The Guangzhou Biobank Cohort Study (2003–8).

		Parental deaths before adulthood (before age of 18 years)					
		Model †	None	One parent		Two parents	
				β	95% CI	β	95% CI
Men	HDL-cholesterol	1	ref	**0.02**	**0.001 to 0.04**	0.03	−0.01 to 0.07
	(mmol/L)	2	ref	0.02	−0.006 to 0.04	0.03	−0.02 to 0.07
				0.01	−0.01 to 0.03	0.01	−0.03 to 0.05
	Waist -ip ratio	1	ref	−0.002	−0.005 to 0.002	**−0.008**	**−0.015 to −0.001**
		2	ref	−0.002	−0.005 to 0.001	**−0.008**	**−0.015 to −0.001**
	White blood cell count*	1	ref	−0.01	−0.09 to 0.09	**−0.30**	**−0.52 to −0.07**
	10^9^/L	2	ref	−0.05	−0.16 to 0.06	**−0.35**	**−0.56 to −0.13**
				−0.04	−0.15 to 0.06	**−0.32**	**−0.54 to −0.11**
Women	HDL-cholesterol	1	ref	−0.01	−0.02 to 0.01	0.01	−0.02 to 0.04
	(mmol/L)	2	ref	−0.01	−0.02 to 0.01	0.01	−0.02 to 0.04
				−0.01	−0.02 to 0.01	−0.002	−0.03 to 0.03
	Waist-hip ratio	1	ref	0.002	−0.0001 to 0.004	−0.001	−0.006 to 0.005
		2	ref	0.0001	−0.003 to 0.002	−0.005	−0.01 to 0.001
	White blood cell count*	1	ref	0.03	−0.04 to 0.09	**0.17**	**0.02 to 0.31**
	10^9^/L	2	ref	−0.02	−0.09 to 0.05	0.07	−0.08 to 0.21
				−0.01	−0.08 to 0.05	0.13	−0.01 to 0.27

†Model 1 adjusted for age.

Model 2 additionally adjusted for father's job type, leg length, education, job type, smoking, use of alcohol and physical activity.

Model 3 additionally adjusted for body mass index and waist-hip ratio.

*only available in recruitment phases 1 and 3 for 5285 men and 14150 women.

**Bold type indicates statistical significance.**

Table 4 shows the association of earliest death of a parent at different ages (0–<3 years, 3–<13 years, 13–<18 years and 18+ years) with the same cardiovascular risk factors, and the validation outcomes. Parental death at 3–<13 years and 13–<18 years was associated with lower seated height and lower 10-word recall score. Adjusted for age, sex, socio-economic position, leg length and lifestyle, parental death at 3–<13 years and 13–<18 years was associated with lower body mass index, but not with the other cardiovascular risk factors, except for an association of parental death at 3–<13 years with lower triglycerides in all three models.

**Table 4:** Adjusted associations of age at earliest death of a parent with cardiovascular risk factors, seated height and delayed 10-word recall score in 28,771 older Chinese men and women in recruitment phases 1, 2 and 3 of The Guangzhou Biobank Cohort Study (2003–8).

		Age at earliest death of a parent						
		18+	13–<18 years		3–<13 years		0–<3 years	
		n = 20819	n = 1846		n = 5000		n = 1106	
	Model †		β	95% CI	β	95% CI	β	95% CI
Systolic blood pressure	1	ref	−0.40	−1.64 to 0.85	0.15	−0.69 to 0.98	0.14	−1.44 to 1.72
(mm Hg)	2	ref	−0.64	−1.88 to 0.60	−0.27	−1.11 to 0.56	−0.27	−1.84 to 1.30
	3	ref	−0.36	−1.56 to 0.83	0.10	−0.71 to 0.91	0.29	−1.23 to 1.81
Diastolic blood pressure	1	ref	−0.31	−0.96 to 0.33	−0.16	−0.59 to 0.27	0.00	−0.82 to 0.82
(mm Hg)	2	ref	−0.39	−1.04 to 0.25	−0.30	−0.73 to 0.13	−0.12	−0.94 to 0.69
	3	ref	−0.24	−0.86 to 0.37	−0.09	−0.51 to 0.33	0.20	−0.58 to 0.99
Fasting plasma glucose	1	ref	0.01	−0.08 to 0.10	−0.01	−0.06 to 0.05	0.02	−0.09 to 0.13
(mmol/L)	2	ref	0.01	−0.08 to 0.09	−0.02	−0.07 to 0.04	0.01	−0.10 to 0.12
	3	ref	0.01	−0.08 to 0.09	−0.003	−0.06 to 0.05	0.03	−0.08 to 0.14
LDL-cholesterol	1	ref	−0.02	−0.05 to 0.02	−0.02	−0.04 to 0.01	−0.003	−0.05 to 0.04
(mmol/L)	2	ref	−0.02	−0.05 to 0.02	−0.01	−0.04 to 0.01	0.001	−0.04 to 0.05
	3	ref	−0.02	−0.05 to 0.02	−0.01	−0.03 to 0.01	0.005	−0.04 to 0.05
HDL-cholesterol	1	ref	−0.002	−0.02 to 0.02	0.01	−0.01 to 0.02	−0.002	−0.03 to 0.03
(mmol/L)	2	ref	−0.001	−0.02 to 0.02	0.01	−0.01 to 0.02	−0.003	−0.03 to 0.02
	3	ref	−0.004	−0.02 to 0.02	0.002	−0.01 to 0.02	−0.01	−0.04 to 0.01
Triglycerides	1	ref	−0.04	−0.12 to 0.05	**−0.06**	**−0.11 to −0.02**	0.04	−0.12 to 0.05
(mmol/L)	2	ref	−0.04	−0.10 to 0.03	**−0.07**	**−0.11 to −0.03**	0.04	−0.12 to 0.04
	3	ref	−0.03	−0.09 to 0.03	**−0.05**	**−0.09 to −0.01**	−0.01	−0.09 to 0.07
Body mass index	1	ref	−0.15	−0.31 to 0.01	**−0.12**	**−0.23 to −0.01**	−0.13	−0.33 to 0.08
	2	ref	**−0.19**	**−0.35 to −0.03**	**−0.19**	**−0.30 to −0.08**	−0.22	−0.42 to 0.01
Waist-hip ratio	1	ref	0.001	−0.002 to 0.004	0.00	−0.002 to 0.002	−0.002	−0.01 to 0.001
	2	ref	0.00	−0.003 to 0.003	−0.002	−0.004 to 0.001	**−0.004**	**−0.01 to −0.001**
White blood cell count*	1	ref	−0.04	−0.13 to 0.05	0.03	−0.03 to 0.10	−0.003	−0.12 to 0.11
10^9^/L	2	ref	−0.06	−0.15 to 0.05	−0.02	−0.08 to 0.05	−0.06	−0.18 to 0.05
	3	ref	−0.05	−0.14 to 0.04	−0.01	−0.07 to 0.05	−0.03	−0.14 to 0.08
Delayed 10-word recall	1	ref	**−0.16**	**−0.25 to −0.07**	**−0.19**	**−0.25 to −0.13**	**−0.21**	**−0.32 to −0.09**
Score	2	ref	**−0.10**	**−0.18 to −0.02**	**−0.09**	**−0.14 to −0.03**	−0.10	−0.20 to 0.01
Seated height	1	ref	**−0.37**	**−0.53 to −0.21**	**−0.40**	**−0.50 to −0.30**	**−0.32**	**−0.52 to −0.12**
(centimeters)	2	ref	**−0.28**	**−0.43 to −0.13**	**−0.24**	**−0.34 to −0.14**	−0.13	−0.32 to 0.06

†Model 1 adjusted for age, sex and the interaction of age and sex.

Model 2 additionally adjusted for father's job type, leg length, education, job type, smoking, use of alcohol and physical activity.

Model 3 additionally adjusted for body mass index and waist-hip ratio.

*only available in recruitment phases 1 and 3 for 5285 men and 14150 women.

**Bold type indicates statistical significance.**

## Discussion

In an under-studied, non-western, developing country a potential marker of early life emotional deprivation, i.e., parental death, was, as expected, associated with lower sitting height [19], and poorer cognition [20], [24]. However, parental death was not clearly associated with cardiovascular risk factors in the expected direction. On the contrary, it was associated with lower body mass index and possibly with lower triglycerides in both sexes and with lower waist-hip ratio and white blood cell count among men.

Although this is a very large study there are some limitations. First, survival bias is possible. If survivorship were an issue we would have expected differences in association by age of which there was no evidence. Second, the infrastructure to facilitate fully representative cohort studies in developing countries such as China is not readily available, which could preclude evidence from a large proportion of the global population in developing countries during a period of transition. Although, this cohort may not be representative, prevalences of relevant morbidities, such as hypertension and diabetes, were similar to those in a recent, representative sample of urban Chinese [21]. Our findings would be biased if people with specific combinations of early life experiences and adult health state were systematically excluded. However, it is not obvious why this should have occurred. Third, we used a simple item, early life parental death, as a measure of emotional adversity. It is possible that parental death is not a source of emotional adversity, perhaps because other family members “stepped in”, or because the effects may be modified by other unmeasured factors, such as resilience or the quality of other relationships. However, parental death had the expected associations with sitting height and cognitive function (Table 2).

Our findings, in a non-western developing country are not consistent with the published evidence to date from developed western populations showing that early life emotional adversity is associated with higher adult cardiovascular risk [5]–[10]. However, a reverse association between type A personality and ischemic heart disease was also recently found in Japan [32], suggesting that some associations may be contextually specific to western populations. Moreover, observations of an association between childhood emotional adversity and adult cardiovascular risk in western populations may be partly due to unhealthy adult behavior, which is associated with both early life emotional adversity and adult cardiovascular risk [33]–[35], whereas our women participants were virtually non-smoking and non-drinking (Table 1). Finally, none of these previous studies [5]–[10] used a counter-example for exposures or outcomes to demonstrate specificity or to quantify potential confounding, although we were only able to use counter-examples for the outcomes (seated height and 10-word recall).

Currently, the pathways by which early life emotional adversity affects long-term cardiovascular health are not completely clear [20], [23], as are the pathways by which contemporaneous adult stress cause cardiovascular disease [36], and the evidence of chronic stress leading to cardiovascular diseases [36]–[40]. Our study from a non-western developing country is most consistent with the simplest explanation, i.e., a lack of positive association between early life emotional deprivation and cardiovascular risk factors.

It is not immediately obvious why early life parental death should be negatively associated with body mass index overall and with waist-hip ratio and white blood cell count among men. It is possible that facing emotional adversity at a young age makes people stronger and more resilient, encourages them to take better care of themselves, helps them to develop healthier attitudes towards adversity or results in them getting more attention from society. However, these influences would be expected to operate similarly for all cardiovascular risk factors. In addition, these influences would also be expected to operate similarly for women as well as men, unless preferential treatment of boys in a traditional Chinese culture countered the detrimental effects of emotional adversity. However, there was no indication of different associations by sex for the validation outcomes.

It is also possible that our observations are due to residual confounding. However, additionally adjusting for several measures of childhood living conditions (model 2 compared with model 1) barely changed the estimates for the cardiovascular outcomes suggesting little such confounding. In contrast, the validation outcomes were attenuated by adjustment for these same confounders, suggesting a potential role. Moreover, residual confounding might have been expected to affect all the cardiovascular outcomes similarly, associations for some outcomes but not others, as we found, suggests that not all observed associations are due to residual confounding. Nevertheless, we have previously observed childhood socio-economic position positively associated with central obesity among men in this population [41], [42], so it is possible that the observed negative association among men of parental death with waist-hip ratio is due to residual confounding by childhood socio-economic position, even though we adjusted for socio-economic position and leg length.

An alternative hypothesis is that the stress of early life emotional adversity may downregulate the gonadotropic axis during growth [22], [43], thus reducing levels of sex-steroids and affecting patterns of fat deposition resulting in a less android body shape among men but a less gynoid body shape among women [44]–[46] with corresponding implications for waist-hip ratio. The gonadotropic axis also interacts with the immune system so the same mechanism would also be expected to have long-term sex-specific effects on the immune system [47]–[49] and thereby inflammatory markers. Specifically, down-regulation of the gonadotropic axis would be expected to result in comparatively better immune function among men, because testosterone suppresses the immune system [48]–[50], but to result in comparatively worse immune function among women, because estrogen promotes immune function [48], [49], [51], consistent with the observed sex-specific effects on white cell count. We do not have information on sex-steroids in this study, so we cannot substantiate this hypothesis further; nevertheless it is a possibility which does explain the specific observed associations.

From a public health perspective, this study indicates the use of evidence from a variety of contexts to inform policy and research priorities Given the importance for prevention of understanding the effect of experiences throughout life, future studies should focus on testing specific biologically based hypotheses, rooted in developmental processes, preferably in populations, such as ours with little association between early life emotional adversity and unhealthy behavior, and little confounding by socio-economic position.

### Conclusions

Our study, from a non-western, developing country population suggests that early life emotional adversity resulting from parental death may sometimes be associated with lower levels of some cardiovascular risk factors particularly among men. Equally importantly, our study demonstrates the need for and role of evidence from different socio-historical contexts in clarifying empirically driven hypotheses from long-term economically developed populations.
